# Increased plasma DYRK1A with aging may protect against neurodegenerative diseases

**DOI:** 10.1038/s41398-023-02419-0

**Published:** 2023-04-04

**Authors:** Jean M. Delabar, Julien Lagarde, Marta Fructuoso, Ammara Mohammad, Michel Bottlaender, Eric Doran, Ira Lott, Isabelle Rivals, Frederic A. Schmitt, Elizabeth Head, Marie Sarazin, Marie-Claude Potier

**Affiliations:** 1 Paris Brain Institute (ICM), Centre National de la Recherche Scientifique (CNRS) UMR 7225, INSERM U1127, Sorbonne Université, Hôpital de la Pitié-Salpêtrière, Paris, 75013 France; 2 Department of Neurology of Memory and Language, GHU Paris Psychiatrie & Neurosciences, Hôpital Sainte Anne, Paris, 75013 France; 3 Paris-Saclay University, BioMaps, Service Hospitalier Frédéric Joliot CEA, CNRS, Inserm, Orsay, 91400 France; 4 School of Medicine, Department of Pediatrics, University of California, Irvine, CA 92697 USA; 5 Equipe de Statistique Appliquée, ESPCI Paris, INSERM, UMRS 1158 Neurophysiologie Respiratoire Expérimentale et Clinique, PSL Research University, Paris, 75005 France; 6 Department of Neurology, University of Kentucky, Lexington, KY 40506 USA; 7 Department of Pathology and Laboratory Medicine, University of California, Irvine, CA 92697 USA

**Keywords:** Biomarkers, Neuroscience

## Abstract

Early markers are needed for more effective prevention of Alzheimer’s disease. We previously showed that individuals with Alzheimer’s disease have decreased plasma DYRK1A levels compared to controls. We assessed DYRK1A in the plasma of cognitively healthy elderly volunteers, individuals with either Alzheimer’s disease (AD), tauopathies or Down syndrome (DS), and in lymphoblastoids from individuals with DS. DYRK1A levels were inversely correlated with brain amyloid β burden in asymptomatic elderly individuals and AD patients. Low DYRK1A levels were also detected in patients with tauopathies. Individuals with DS had higher DYRK1A levels than controls, although levels were lower in individuals with DS and with dementia. These data suggest that plasma DYRK1A levels could be used for early detection of at risk individuals of AD and for early detection of AD. We hypothesize that lack of increase of DYRK1A at middle age (40–50 years) could be a warning before the cognitive decline, reflecting increased risk for AD.

## Introduction

The complex pathologic cascade that leads to Alzheimer’s disease (AD) begins decades before clinical symptoms develop [1–3]. This suggests that effective prevention will require predicting who will develop AD long before the onset of symptoms. Therefore, there is significant interest in identifying biomarkers for individuals at increased risk of AD, who can then be targeted with preventive interventions such as risk factor reduction, behavioural modification, and pharmacologic treatment [4, 5].

The kinase DYRK1A is encoded on HSA21 in 21q22.2 [6, 7]. The gene comprises 151 kb and 15 exons (Ensemble release 90); it encodes two main protein isoforms of 763 and 754 amino acids. DYRK1A autophosphorylates on tyrosine, serine, and threonine residues but phosphorylates its substrates only on serine and threonine residues. The large number of substrates phosphorylated by DYRK1A and the wide range of interacting partners indicate that DYRK1A is capable of controlling a variety of molecular processes. These processes underlie several physiological functions at different stages of life: during neurogenesis at early development, in neuronal plasticity during brain functioning, and during aging [8]. Our previous work showed that plasma from AD patients has significantly lower DYRK1A levels compared to controls [9, 10]. We also recently found that plasma DYRK1A can be used to identify elderly people with subjective memory complaint who are at risk for brain amyloid β (Aβ) deposits [11]. In mouse models, our work also showed increased DYRK1A levels in the brain of older wild-type mice when comparing 4-month-old with 12- and 17-month-old mice [12]. Similarly, our results in humans also indicate an age-dependent increase of plasma DYRK1A levels, which might be protective for aging and cognitive decline [11].

DYRK1A may participate in different pathways that change over time, with an initial role during development and then other roles during adulthood and aging, and may differ across tissue types. *DYRK1A* is located on chromosome 21, suggesting this gene may be overexpressed in cells from individuals with Down syndrome (DS). *DYRK1A* transcripts from DS lymphoblastoids are overexpressed 1.4-fold [13] compared to individuals without DS. Similar overexpression is observed in mouse models with three copies of *Dyrk1a* [14, 15]. The brains of AD patients [16] and mouse models of AD with cognitive impairment show increased DYRK1A levels [17, 18], which are associated with increased Tau phosphorylation that can be reversed with DYRK1A inhibitors [19]. Moreover, in other mouse models with hyperhomocysteinemia-induced increased liver DYRK1A, brain DYRK1A levels are decreased [20]. Collectively, these results suggest that low plasma levels of DYRK1A may be a risk factor for AD in the early stages of the disease [9, 10].

Therefore, this study tested our previous observations by analysing plasma samples from the SENIOR cohort of cognitively healthy elderly volunteers [21] and the SHATAU7-IMATAU cohort of patients with AD pathology [22, 23]. We also compared levels of plasma DYRK1A from patients with various tauopathies in the SHATAU7-IMATAU cohort: frontotemporal lobar degeneration, hippocampal sclerosis, and progressive supranuclear palsy and corticobasal degeneration. Individuals with DS develop dementia earlier (at 40–60 years of age) than the general population, with half of DS individuals affected by 55 years of age [24, 25]. Thus, we also assessed DYRK1A levels in plasma samples from individuals with DS in a longitudinal cohort from the University of Kentucky [26].

## Materials and methods

### Young controls

Plasma samples from young individuals were obtained from Cambridge BioScience Ltd. (9 Caucasians, 7 Asians, 1 Black, 3 Mixed), from blood samples collected on heparin; these samples were compared to SENIOR and SHATAU7-IMATAU cohorts (Table 1) [11].

**Table 1:** Demographic and clinical data of studied groups.

	Number of subjects	Female-Male	Age (years)- Range	MMSE	DYRK1A (ng/ml) (SEM)	PIB-PET (SEM)	Tau-PET	E4/E4E4
young controls								
	20	6-14	25.5- (19–50)		1.64 (0.15)			
middle aged controls								
	19	12-7	47.5- (36–64)		2.45 (0.23)			
cohort SENIOR	96	48-48	59	29.2				
low amyloid	28	9-18	59- (50–69)	29.1	4.12 (0.34)	1.18 (0.013)		(4/0)
high amyloid	49	27-21	60- (50–69)	29.3	2.81 (0.18)*	1.34 (0.021)****		(9/1)
cohort SHATAU7-IMATAU								
AD	43	25-18	67.4- (53–87)	20	1.7 (0.1)	2.79****	2.7****(0,2)	(17/4)
HS	27	9-18	74.8- (41–85)	22.5	1.57 (0.13)	1.7	1.33 (0,05)	(4/1)
FTLD	23	10-13	70.5- (55–78)	17.8	1.42 (0.1)	1.32	1.28 (0,06)	(4/1)
PSP-CBD	9	7-2	65.8- (58–79)	23	2.11 (0.17)	1.45	1.22 (0,1)	(4/0)
cohort DS LCLs								
CTRL	12	6-6	45.6		100			
DS	8	3-5	52.7		180~ (11.6)			
DS-AD	8	3-5	49.7		138~ (10.9)			
cohort DS plasmas								
DS	38	18-19	38.5- (25–49)	91^a^	7.34 (0.37)			(9/2)
DS-AD	32	22-10	51- (31–65)	80^a^	3.99 (0.31)			(7/0)

^a^*SIB*, Severe impairment battery. *MMSE* Mini Mental State Examination, *PiB-PET* Pittsburgh Compound-B positron emission tomography, *Tau-PET* Tau protein positron emission tomography; *AD* Alzheimer’s disease, HS, hippocampal sclerosis,*FTLD* frontotemporal lobar degeneration, *PSP-CBD* progressive supranuclear palsy and corticobasal degeneration, *LCL* lymphoblastoid cell line, ~arbitrary units, *DS* Down syndrome without dementia, *DSAD* Down syndrome with dementia. *P < 0.05, ****P < 0.0001

### Middle-aged controls

Blood samples from parents of individuals with Down syndrome (Aneuploidy program [13]) have been collected on sodium citrate and collected plasmas were used to compare with patient cohorts.

### SENIOR cohort

The SENIOR cohort is a group of cognitively healthy volunteers aged 50–70 years at the time of inclusion who agreed to annual examinations over 10 years [21]. In March 2012, the NeuroSpin Center in Saclay, France, initially contacted 300 individuals who indicated interest in participating in the study after public advertisement via flyers and invitations sent to former study participants. Among these volunteers, 186 subjects reporting no memory complaints, uncontrolled chronic diseases, and/or MRI incompatibility were invited for further neuropsychological assessment and neuroimaging via 3 T MRI for final screening. A total of 142 subjects were included and completed the baseline examination. Forty-four subjects were excluded for the following reasons: failed neuropsychological tests, detected structural abnormalities on MRI, moved during MRI imaging, did not meet inclusion criteria, or experienced discomfort during the imaging session. Blood samples were collected on heparin and processed within 2 h to prepare plasmas.

To measure the cerebral Aβ load of participants, cerebral PET was performed at Service Hospitalier Frédéric Joliot (Orsay, France) on a high-resolution neuroimaging tomograph (Siemens Healthineers). Aβ-PET dynamic acquisition was performed 40–60 min after injection of 341 ± 68 MBq of [11 C]-Pittsburgh Compound-B (PiB). All corrections (attenuation, normalization, random and scatter coincidences) were incorporated in an iterative ordered-subset expectation maximization reconstruction. Segmentation into cerebral regions of interests and cortical thickness was performed using the Killiany/Desikan parcellation atlas. Aβ-PET imaging analysis was performed as previously described [27, 28]. Parametric images were created using BrainVisa software.

### SHATAU7-IMATAU cohort

We included 98 participants from the SHATAU7-IMATAU study (NCT02576821-EudraCT2015-000257-20) recruited during March 2016–November 2019. The Ethics Committee (Comité de Protection des Personnes Ile-de-France VI) approved the studies. All subjects provided written informed consent. Fourty three patients diagnosed with AD at mild cognitive impairment or mild dementia stage were included according to the following criteria: (i) cerebrospinal fluid biomarker profile suggestive of AD [total tau/Aβ (Aβ42) > 0.52, which provided 93% sensitivity and 83% specificity in a previous publication [29] (ii) [11 C]-PiB-PET Global Cortical Index score of >1.4515 ^27^16; and (iii) Clinical Dementia Rating score of ≤1. Of the 43 participants, 29 took cholinesterase inhibitors, and two of these also took N-methyl-D-aspartate antagonists. During the two-year follow-up, treatment changes were limited: cholinesterase inhibitors were discontinued in three patients and were introduced in three other patients. All participants underwent complete clinical and neuropsychological assessments, 3 T brain MRI, and [11 C]-PiB and [18 F]-flortaucipir PET imaging at baseline. The SHATAU7-IMATAU study included also individuals with non-AD pathologies classified according to the results of imaging studies as frontotemporal dementia (22), hippocampal sclerosis (25), and progressive supranuclear palsy/corticobasal degeneration (8). The blood samples were collected from participants into citrate containers, and the containers were immediately placed on ice until processed. Plasma was obtained by centrifugation of containers for 15 min at 2000 g at 4 °C, then rapidly frozen and stored at − 80 °C until analysis.

### Kentucky cohort

The Kentucky cohort includes individuals with DS with a wide age range (25–64 years) to detect early cognitive changes. Inclusion criteria were: (i) existing diagnosis of DS, (ii) >25 years old, (iii) medically stable, (iv) completion of annual visits with MRI and blood samples, (v) English speaking, and (vi) able to tolerate MRI. Exclusion criteria were: (i) not medically stable and have changed medications in the last 3 months (with the exception of anxiolytic use as needed for medical procedures), and (ii) diagnosis of neurological disease other than DS. Research procedures were independently reviewed and approved by the University of Kentucky Institutional Review Board. Participants provided informed consent or assent with guardian approval. Participants were community-residing men and women with DS recruited through local DS support groups and residential facilities in Kentucky, southern Indiana, and southern Ohio. Cognitive assessment was performed longitudinally using the Brief Praxis Test [30], Severe Impairment Battery [31], and Dementia Scale for People with Learning Disabilities [32, 26]. Blood samples were collected on EDTA and rapidly processed to isolate plasmas which were stored at −80 °C.

### Lymphoblastoids cohort

An EBV immortalization protocol was used to establish LCLs [13, 33]. DS and DSAD blood samples were obtained during patient examinations and were derived from subjects with confirmed full trisomy 21. Control blood samples were obtained from parents of individuals with DS described during the AnEUploidy study and were aged matched with DS individuals [13]. Cognitive assessment was performed longitudinally using the Severe Impairment Battery [31]. All subjects were in the age range of 40 to 60 years. Results are reported as a combination of male and female LCLs for each group.

### Immunometric tests

Immunoassay plates were spotted with biotinylated AC4 (from a set of seven monoclonal antibodies raised against a short form of DYRK1A, 1–502 aa) on MSD GOLD Small Spot Streptavidin 96-well plates (Meso Scale Diagnostics, Rockville, MD, USA). Spotting was performed by the Meso Scale Diagnostics spotting facility. After incubation with plasma samples or calibrator samples (serial dilution of DYRK1A), MSD GOLD SULFO-TAG conjugated detection antibody (AC6) was used to quantify DYRK1A protein levels on a MESO QuickPlex SQ120 instrument (Meso Scale Diagnostics) using electrochemiluminescence detection. AC4 and AC6 were already used in a previous study (9) Plasma samples underwent a single freeze–thaw cycle before analyses. All samples were measured in duplicate with a coefficient of variability acceptance criteria of <20%, and within one round of experiments with the same batch of precoated plates. Baseline and longitudinal samples obtained from each participant were measured side by side in the same run to avoid the effect of run-to-run variability. All analyses were performed by one technician, who was blind to clinical diagnosis. Concentrations are shown in ng/ml.

### Statistical analysis

Data are presented as mean ± standard deviation or median with interquartile range. D’Agostino and Pearson omnibus normality test was used for all data, and Mann-Whitney U-test was used for comparisons between groups. Correlations between DYRK1A levels and standardized uptake value ratio (SUVR) were assessed for each marker using Spearman’s (nonparametric) test. *P* < .05 for intergroup comparisons and *P* < .01 for correlations were considered statistically significant. Correlations between DYRK1A levels and age were assessed using Pearson’s test. Graphs were prepared with GraphPad Prism software (version 6, La Jolla, CA, USA).

## Results

### SENIOR cohort

**Fig. 1:**
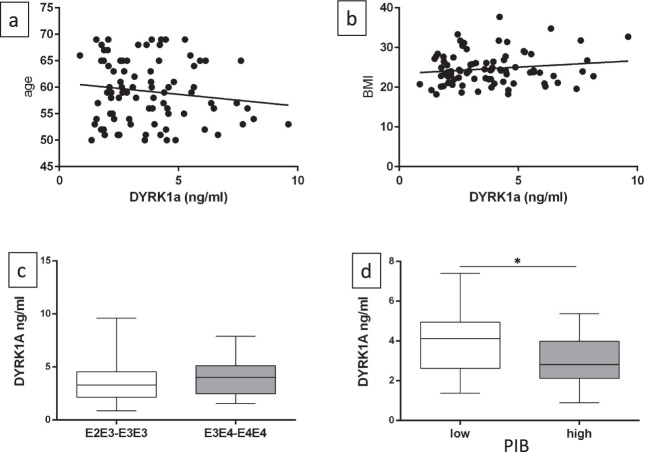
Characterisation of plasma DYRK1A levels for the SENIOR cohort. **a** Correlation between DYRK1A levels and age. **b** Correlation between DYRK1A levels and body mass index (BMI). **c** DYRK1A levels stratified by *APOE* genotype. **d** DYRK1A levels stratified by PiB-PET SUVR with a cut-off at 1.235. Graph bars indicate mean ± standard error of the mean; *P < .05. PiB-PET SUVR, Pittsburgh Compound-B positron emission tomography standardized uptake value ratio.

Using our improved sandwich ELISA technique(Methods section), we assessed DYRK1A levels in plasma samples from the SENIOR cohort (Table 1) using two standardization methods: a standard curve with varying amounts of synthetic DYRK1A-derived peptide, and plasma from 10 young control individuals (~26 years). We observed no correlation between DYRK1A levels and age (Fig. 1a) or between DYRK1A levels and body mass index (BMI) (Fig. 1b). When stratified by *APOE* genotype, SENIOR participants were distributed among genotypes (E3E4 and E4E4 for *APOE ε4* carriers and E2E3 and E3E3 for non *APOE ε4* carriers, and average DYRK1A levels were similar in individuals with or without the *APOE ε4* allele (Fig. 1c). We then stratified low and high PiB-PET SUVR with different cut-off values (PiB-PET SUVR = 1.2–1.3). We found a significant difference for a cut-off of 1.235, corresponding to DYRK1A levels of 4.1 ng/mL for the low amyloid group and 2.8 ng/mL for the high amyloid group (*P* = 0.02) (Fig. 1d). Comparison of the four control groups young ctrl, middle aged ctrl, SENIOR with a low PIB-PET (LP), SENIOR with a high PIB-PET (HP) is reported in Table 2.

**Table 2:** Combined effects of age, gene dosage and dysregulation of DYRK1A on ratios of DYRK1A plasma levels.

		Young	Middle aged	cog healthy HP	cog healthy LP	AD	DS	DSAD
	ng/ml	1.64	2.45	2.42	3.92	1.7	7.34	3.99
Young	1.64		1.49	1.47	2.39	1.03	4.47	2.43
Middle aged	2.45			0.98	1.6	0.69	2.99	1.62
cog healthy HP	2.42				1.62	0.4	3.03	1.64
cog healthy LP	3.92					0.43	1.87	1.01
AD	1.7						4.31	2.34
DS	7.34							0.54

### SHATAU7-IMATAU cohort

**Fig. 2:**
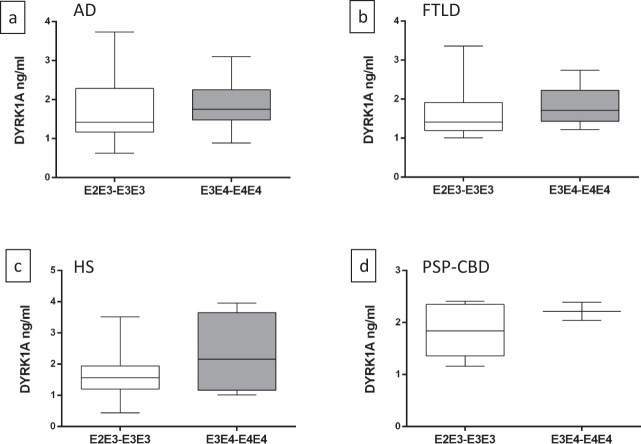
Plasma DYRK1A levels stratified according to *APOE* genotype for patients. **a** Alzheimer’s disease, **b** frontotemporal lobar degeneration, **c** hippocampal sclerosis, or **d** progressive supranuclear palsy and corticobasal degeneration.

The SHATAU7-IMATAU cohort includes patients with four neurological diseases (Table 1): AD, frontotemporal dementia, hippocampal sclerosis, and progressive supranuclear palsy/corticobasal degeneration. When stratified by *APOE* genotype, average DYRK1A levels were similar across individuals with all four diseases (Fig. 2).

**Fig. 3:**
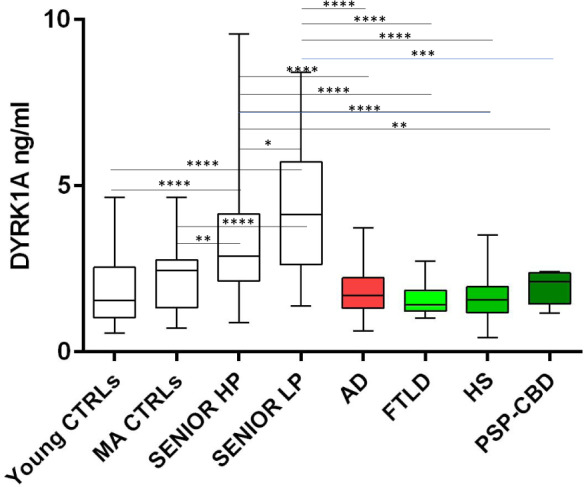
Plasma DYRK1A protein levels for the young control cohort, the middle aged control cohort, the SENIOR control cohort with low (LP) and high (HP) PiB-PET SUVR (cut-off: 1.235) and for the SHATAU7-IMATAU cohort of Alzheimer’s disease (AD), frontotemporal lobar degeneration (FTLD), hippocampal sclerosis (HS), or progressive supranuclear palsy and corticobasal degeneration (PSP-CBD) patients. *P < .05; ***P < .001; ****P < .0001. PiB-PET SUVR, Pittsburgh Compound-B positron emission tomography standardized uptake value ratio.

**Fig. 4:**
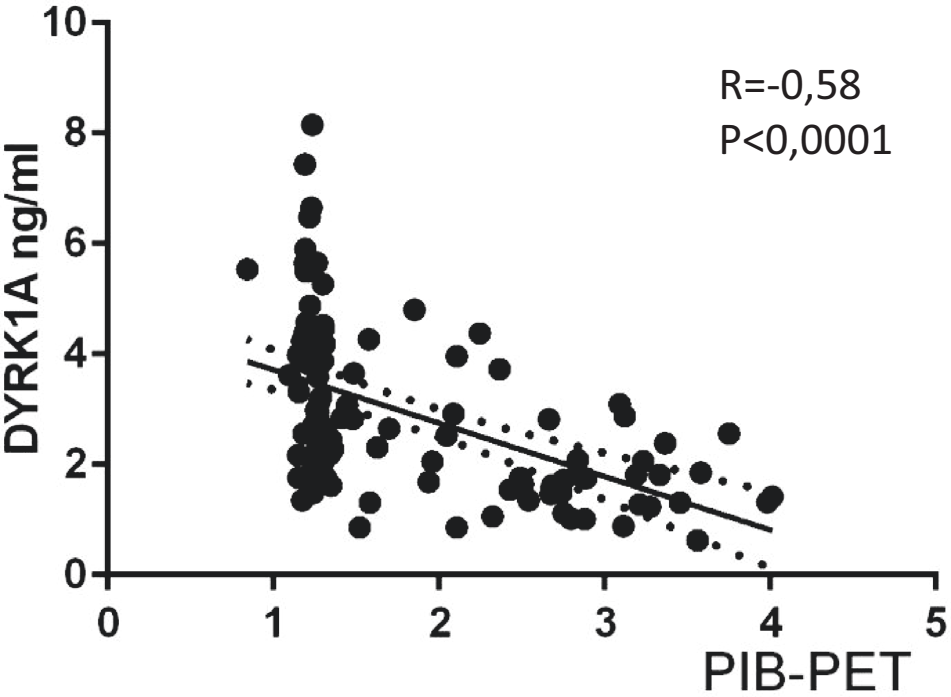
Correlation analysis between plasma DYRK1A levels and PiB-PET SUVR levels for SENIOR cohort and SHATAU7-IMATAU cohort of AD. Correlation was assessed with nonparametric Spearman’s rank test. Graph shows regression lines with 95% confidence interval. PiB-PET SUVR, Pittsburgh Compound-B positron emission tomography standardized uptake value ratio.

No correlation was found between DYRK1A and AD cerebrospinal fluid biomarkers such as Tau, pTau, and Aβ42. We compared DYRK1A levels in control groups, including a cohort of young controls, a cohort of middled aged individuals and the SENIOR cohort with high and low brain Aβ deposits from the SENIOR cohort (HP and LP, respectively) with the four disease groups of the SHATAU7-IMATAU cohort. Young controls were significantly different from the two SENIOR subgroups. Middle-aged controls were not significantly different from the HP SENIOR subgroup. The four SHATAU7-IMATAU disease subgroups differed significantly from the two SENIOR subgroups with the exception of the progressive supranuclear palsy/corticobasal degeneration group, which was not significantly different from the high-PiB-PET SUVR SENIOR subgroup. The four SHATAU7-IMATAU disease subgroups did not differ significantly from the group of young controls (Fig. 3). We also identified significant negative correlation between plasma DYRK1A levels and PiB-PET SUVR for the AD (SHATAU7-IMATAU) and SENIOR cohorts (*P* < 0.0001) (Fig. 4).

### Age effect in control groups

**Fig. 5:**
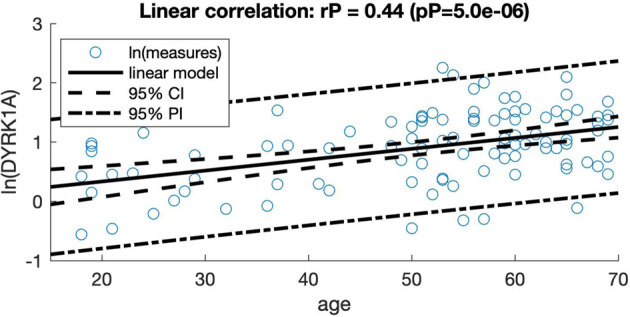
Correlation analysis between age and plasma DYRK1A levels (ln) for control groups including the young control group, the middle aged control group, the SENIOR cohort (LP and HP). Graph shows regression lines with 95% confidence interval (CI) and 95% prediction interval (PI).

Global analysis of the four control groups (young, middle-aged and SENIOR subgroups) revealed a positive correlation between age and plasma DYRK1A levels (pP= 5 e^−06^) (Fig. 5).

### Lymphoblastoids from individuals with DS

**Fig. 6:**
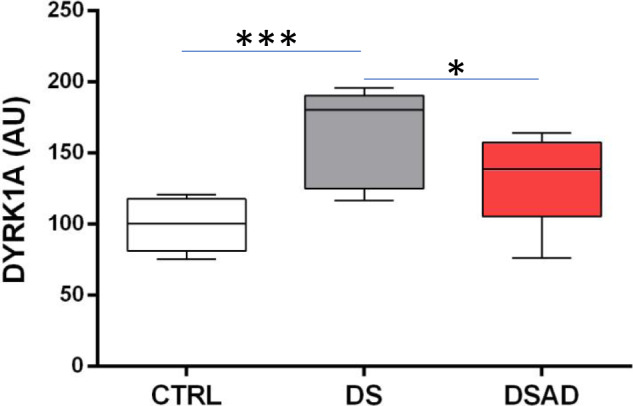
DYRK1A levels from lymphoblastoid cell lines (LCLs) generated from controls (CTRL), individuals with Down syndrome (DS), and individuals with Down syndrome and dementia (DSAD). AU arbitrary units, *P < 0.05; ***P < 0.001.

Lymphoblastoid cell lines established from lymphocytes of individuals with DS without dementia (DS), individuals with DS and with dementia (DSAD), and age-matched controls were grown in standard medium, and proteins were isolated to quantify DYRK1A. Relative DYRK1A expression in DS lymphoblastoids was significantly increased compared to controls (DS/CTRL = 1.8; *P* = 0.0002), while DYRK1A expression in DSAD lymphoblastoids was significantly decreased compared to DS (DS/DSAD = 1.3; *P* = 0.04) but not significantly different from controls (Fig. 6).

### Kentucky cohort

**Fig. 7:**
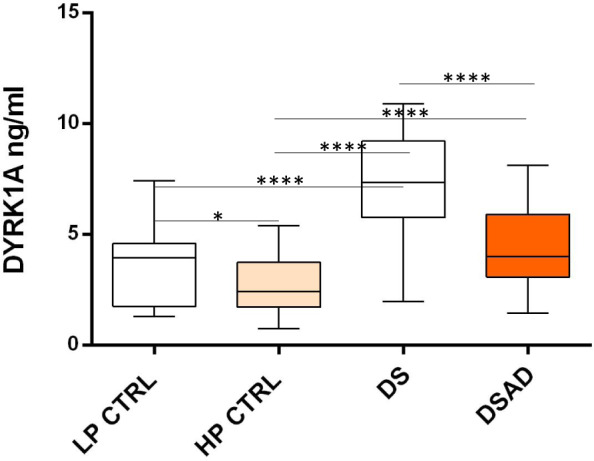
Plasma DYRK1A levels for the SENIOR cohort (LP and HP) and for the Kentucky cohort of participants with Down syndrome (DS) and participants with Down syndrome and dementia (DSAD). *P < 0.05, ****P < 0.0001.

We measured DYRK1A in 230 plasma samples collected longitudinally over 4 years from 70 individuals with DS, DSAD, or cognitive regression. Individuals who exhibited cognitive regression were included in the DSAD group, and DYRK1A values in various samples from the same individual were averaged. DYRK1A levels were significantly lower in the DSAD compared to DS group (DS/DSAD = 1.84; *P* < 0.0001) (Fig. 7). Comparison with the low Aβ SENIOR group as a control yielded a DS/CTRL ratio of 1.78, while comparison with the high Aβ SENIOR group yielded a DS/CTRL ratio of 1.42 (Table 2).

## Discussion

In the whole SENIOR cohort, DYRK1A levels were quite disperse. However when stratifying with PiB-PET SUVR, the group with low Aβ had significantly higher DYRK1A than the group with high Aβ, indicating that individuals with low DYRK1A are potentially at-risk for dementia-associated pathologies such as brain Aβ deposition. DYRK1A levels were not correlated with age in the SENIOR cohort, suggesting that the increase of DYRK1A occurred before the age of 60. We observed no correlation with BMI, which was previously reported as a risk factor for dementia [34].

These results are reminiscent of our findings with the INSIGHT cohort of memory complainers without clinical signs of cognitive decline, for which we also observed an association between low DYRK1A levels and high Aβ deposits in the brain [11]. Individuals with low Aβ load also had higher DYRK1A levels than young individuals, indicating a potential increase of plasma DYRK1A during aging as individuals with high Aβ load had DYRK1A levels similar to young individuals. This paralleled our previous observations of increased Dyrk1A levels in the brain of wild-type aging mice (4, 12, and 17 months), and also in Dyrk1A transgenic mice even though these mice already overexpress Dyrk1A [12]. Increase of DYRK1A is already present in middle age mice (12 months old). In human, this increase could be observed at 40–50 years, younger than the age of the SENIOR cohort. When a correlation analysis is performed on DYRK1A levels and age for the four control groups, we show a strong correlation which allow to define confidence and prediction intervals (Fig. 5). In addition, lower DYRK1A levels were present in AD and in other dementia-associated pathologies, including frontotemporal lobar degeneration, hippocampal sclerosis, or progressive supranuclear palsy/corticobasal degeneration. We thus hypothesize that age-dependant increase of DYRK1A occurs with normal aging and either leads or is associated to a lower risk of brain Aβ pathology as observed in the SENIOR cohort. Individuals from the SHATAU7-IMATAU cohort had DYRK1A levels significantly lower than the elderly control group with low PiB-PET SUVR and similar to our young control group. This difference was not observed when comparing the PSP-CBD group with the control group with high PIB-PET-SUVR, suggesting that low DYRK1A level is not a strong risk marker for PSP-CBD.

A potential limitation of this study is linked to blood collection performed in three different types of tubes: however when comparing cohorts of different age groups (young on heparin, middle aged on citrate and aged on heparin) we observed a linear correlation; moreover a low level of DYRK1A is observed in AD patients (on citrate) compared to aged controls (on heparin) and in DSAD patients (on EDTA) compared to DS patients (on EDTA) suggesting that DYRK1A changes associated with dementia are not caused by an effect of blood types of tube.

The decrease of DYRK1A could be due to truncation or degradation of full-length DYRK1A. In 2015, Jin et al, [35] hypothesized that calpain truncates and activates DYRK1A. The authors’ analyses of brain samples revealed that bands characteristic of DYRK1A at 95 kDa appeared at a lower molecular weight in AD patients than in controls, suggesting that DYRK1A truncation occurs in AD. Yet the bands expected to be degradation or truncation products, were not increased in AD patients, indicating that the antibody the authors used might cross-hybridise with other molecular species. Nevertheless, our DYRK1A immunoassay is detecting full-length as well as truncated protein.

We then investigated DYRK1A in a group of individuals with DS. These individuals develop neuropathological features of AD at >40 years old, and half of DS patients present cognitive impairment indicative of dementia by 55 years of age [24, 25].

**Fig. 8:**
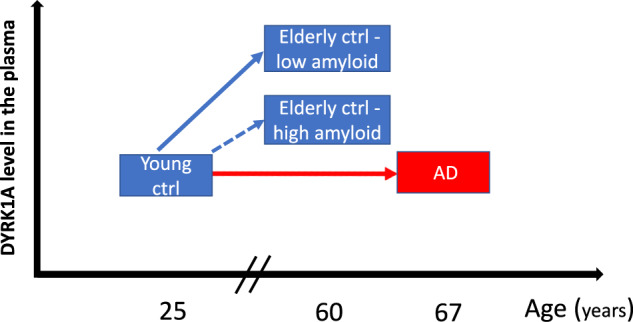
Schematic of changes in plasma DYRK1A levels during aging in controls and in individuals with AD.

From studies on individuals without dementia and without known genetic abnormalities (INSIGHT and SENIOR cohorts), we hypothesized that diploid control individuals with low Aβ deposition would have increasing DYRK1A levels with age (40–60 years old) (Table 2) and might be considered at lower risk of developing dementia (Fig. 8). This critical period might correspond to 20–40 years of age for individuals with DS. Our results with cultured lymphoblastoids showed lower DYRK1A levels in the DSAD group compared to DS group. Lymphoblastoids are established cell lines, indicating that genetic or epigenetic regulation of DYRK1A levels was present before transformation of the cells. Further, plasma from a longitudinal cohort of DS and DSAD individuals showed significant differences between groups, although both DS and DSAD had strongly increased DYRK1A levels compared to controls of a similar age (46 years for the middle aged control group) (Table 2). The DS group had a 2.99-fold increase of DYRK1A (Table 2), which is higher than the expected value of 1.5 for trisomy. This elevated ratio may be associated with accelerated aging in DS, thus combining the effect of trisomy (ie: x1.5) with the effect of aging (ie: x2).

Psychometric data from a cohort of 445 individuals with DS indicate that a single-point assessment of acquired mild cognitive impairment, which is expected for the majority of adults with DS, reveals two peaks for age‐related prevalence of impairment, suggesting that the risk for AD onset conferred by DS is moderated by other factors than trisomy [36], which could include the *APOE ε4* allele or overexpression of regulatory factors encoded by HSA21 genes. Increased DYRK1A could have contradictory effects related to AD: an anti-inflammatory and protective effect [37–39] associated with low homocysteine levels in the periphery as well as a deleterious hyperphosphorylation of Tau protein in the brain [17, 40]. Liver treatment with an adenovirus expressing Dyrk1A normalizes hepatic DYRK1A level and decreases hyperhomocysteinemia in mice with hyperhomocysteinemia. AAV-mediated hepatic Dyrk1A gene transfer increases DYRK1A protein level in the periphery and decreases DYRK1A level in the brain of hyperhomocysteinemic mice [20]. Hyperhomocysteinemia is associated with low DYRK1A levels [41] in the periphery and with a change in inflammatory status [42, 43]. Increased plasma DYRK1A levels with aging may exert an anti-inflammatory effect at the beginning of the neuropathological process, thus delaying early signs of neurodegeneration and dementia. Conversely, low plasma DYRK1A levels may be associated with vulnerability to AD and AD-related pathologies. Accordingly, controlling DYRK1A levels during aging may facilitate preventive intervention. However, we cannot exclude the possibility that lack of increase of DYRK1A during aging could be due to modifications of regulatory processes in other pathways involved in neurodegeneration. In that case, DYRK1A levels would be a marker of these alterations, and acting upon pathways regulating DYRK1A and other factors would be a therapeutic target.

Aging is associated with increased risk of dementia in individuals with DS, with a mean age of diagnosis of 55 years [24, 25]. However, a fraction of individuals with DS will not develop dementia or develop it later (>55–60 years old). Our work suggests that the critical point for dementia is not DYRK1A levels alone but a ratio between the levels of DYRK1A and another HSA21 gene that we call *X*. High DYRK1A/*X* ratio may delay onset of AD-type dementia in DS. The increased risk for dementia in individuals with DS is associated with trisomy of the *APP* gene [44]. In addition, *APP* locus duplications cause autosomal dominant early-onset AD [45]. *APP* could thus be a good candidate for *X*. A low DYRK1A/APP ratio could confer high risk of AD. This hypothesis would be also applicable to diploid elderly individuals with high DYRK1A levels and low AD risk, as these individuals have a high DYRK1A/*X* ratio, while diploid elderly individuals with low DYRK1A and AD dementia have a low DYRK1A/*X* ratio.

Our results show that low plasma DYRK1A may indicate at-risk individuals who may benefit from early treatment to prevent AD. Further experiments with genetically engineered mouse models of AD with 1 or 3 copies of *Dyrk1A* may help unravel the effects of DYRK1A. Additional longitudinal human cohorts are also needed to confirm these findings and determine the timeline of DYRK1A variation compared to Aβ changes.
